# Production and characterization of rhamnolipids by *Pseudomonas aeruginosa* isolated in the Amazon region, and potential antiviral, antitumor, and antimicrobial activity

**DOI:** 10.1038/s41598-024-54828-w

**Published:** 2024-03-12

**Authors:** Sidnei Cerqueira dos Santos, Chayenna Araújo Torquato, Darlisson de Alexandria Santos, Alexandre Orsato, Karoline Leite, Juliana Mara Serpeloni, Roberta Losi-Guembarovski, Erica Romão Pereira, André Luiz Dyna, Mario Gabriel Lopes Barboza, Matheus Hideki Fernandes Arakawa, José Augusto Pires Bitencourt, Sebastião da Cruz Silva, Giulian César da Silva Sá, Pamela Dias Rodrigues, Cristina Maria Quintella, Lígia Carla Faccin-Galhardi

**Affiliations:** 1 Biology College, Federal University of Southern and Southeast Pará (Unifesspa), Marabá, PA 68500-000 Brazil; 2 Chemistry College, Federal University of Southern and Southeast Pará (Unifesspa), Marabá, PA 68500-000 Brazil; 3 Department of Fundamental Chemistry, Federal University of Pernambuco (UFPE), Recife, PE 50740-560 Brazil; 4 Department of Chemistry, State University of Londrina (UEL), Londrina, PR 86057-970 Brazil; 5 Department of General Biology, State University of Londrina (UEL), Londrina, PR 86057-970 Brazil; 6 Department of Microbiology, State University of Londrina (UEL), Londrina, PR 86057-970 Brazil; 7 Vale Technological Institute (ITV), Belém, PA 66055-090 Brazil; 8 Department of General and Inorganic Chemistry, Federal University of Bahia (UFBA), Salvador, BA 40170-115 Brazil

**Keywords:** Greener technology, Microbial process, *Pseudomonas aeruginosa*, Rhamnolipid, Biochemistry, Biotechnology, Microbiology

## Abstract

Biosurfactants encompass structurally and chemically diverse molecules with surface active properties, and a broad industrial deployment, including pharmaceuticals. The interest is growing mainly for the low toxicity, biodegradability, and production from renewable sources. In this work, the optimized biosurfactant production by *Pseudomonas aeruginosa* BM02, isolated from the soil of a mining area in the Brazilian Amazon region was assessed, in addition to its antiviral, antitumor, and antimicrobial activities. The optimal conditions for biosurfactant production were determined using a factorial design, which showed the best yield (2.28 mg/mL) at 25 °C, pH 5, and 1% glycerol. The biosurfactant obtained was characterized as a mixture of rhamnolipids with virucidal properties against Herpes Simplex Virus, Coronavirus, and Respiratory Syncytial Virus, in addition to antimicrobial properties against Gram-positive bacteria (*Staphylococcus aureus* and *Enterococcus faecium*), at 50 µg/mL. The antitumor activity of BS (12.5 µg/mL) was also demonstrated, with potential selectivity in reducing the proliferation of breast tumor cells, after 1 min of exposure. These results demonstrate the importance of studying the interconnection between cultivation conditions and properties of industrially important compounds, such as rhamnolipid-type biosurfactant from *P. aeruginosa* BM02, a promising and sustainable alternative in the development of new antiviral, antitumor, and antimicrobial prototypes.

## Introduction

Biosurfactants (BS) are amphipathic molecules, produced mainly by microorganisms, capable of reducing surface and interfacial tensions at the air–water and oil–water interfaces, respectively, in addition to having emulsion-forming properties^1^. Biosurfactants show structural diversity of surface-active substances and are classified, according to the chemical composition and microbial origin, into glycolipids, lipopeptides, lipoproteins, fatty acids, neutral lipids, phospholipids, polymeric, and particulate surfactants^2^. Among them, glycolipids have been widely studied due to their excellent emulsification properties, especially rhamnolipids (RL) produced by the bacteria *Pseudomonas aeruginosa*^3^, one of the most studied bacterial producers of biosurfactants. This ubiquitous bacterium can be found in different environments and can also be advantageously used in biotechnological applications^4^.

BS has been an environmentally acceptable alternative for numerous applications, as it is produced from renewable resources, has low toxicity, and presents an attractive yield under extreme conditions of pH, temperature, and salinity. Furthermore, the value global BS (rhamnolipid) market was US$ 8.45 billion in 2022, with a projection growth of 5% (US$ 14.3 billion) by 2032^5^. However, the optimization of BS production can be a time-consuming process when individual factors are assessed separately^4^. To make BS production economically feasible, studies have been carried out to optimize the factors that influence its production, ensuring optimum conditions for microbial growth and BS yield^5^. Among the main concerns, the use of bacterial strains notoriously efficient in BS production and the characteristics of microbial cultivation, such as the composition of the culture medium, supply of carbon and nitrogen sources, balance of pH, and temperature, stand out^6^. Considering factorial design in BS production also ensures efficient performance, since factorial design is a statistical tool capable of establishing and quantitatively assessing the factors that are more relevant in the response of interest^7^.

Biosurfactant produced by *P. aeruginosa* have excellent physicochemical characteristics, in addition to presenting low toxicity, and high biodegradability and yield, compared with other bacterial BSs^8^. Several biological activities are described for BS produced by *P. aeruginosa*, including antiviral, antitumor, and antimicrobial activities^2,9^, suggesting its indication as potential prophylactic and/or therapeutic adjuvant. The discovery of new, safe, and effective broad-spectrum antitumor and antimicrobial/antiviral agents is essential. In this context, biosurfactants are promising alternatives in developing these agents as prophylactic and/or therapeutic. The present work assessed the optimized production of biosurfactant obtained by bacteria isolated from the soil of a mining area in the Amazon region (Brazil), in addition to investigating its antiviral, antitumor, and antimicrobial activities. Brazil is one of the largest ore producers in the world, mainly iron and bauxite, with the Eastern Amazon and specifically the state of Pará being home to the largest deposits of these ores^10^. Therefore, the discovery and characterization of a new molecule from a mining area is very promising due to the influence of the environment on the microorganism physiology, in addition to expanding the literature with relevant pharmacological compounds, considering the limitations in available therapies.

## Material and methods

### BS production

#### Isolation, DNA extraction, sequencing, and molecular identification

The bacterium, formerly BM02, was isolated from the soil of a bauxite mine, in the regeneration process, located in Paragominas-PA, Brazil. This soil has an acidic pH (4.4–5.9), aluminum content (up to 2.3 cmolc/dm^3^) and saturation reaching 95%, and the occurrence of calcium (up to 2.3 cmolc/dm^3^), magnesium (up to 1.1 cmolc/dm^3^), and potassium (up to 0.1 cmolc/dm^3^) ions^11^. BM02 was preserved (− 20 °C) in a glycerol solution at the Bio-test and Bio-processes Laboratory of Unifesspa, Marabá-PA, Brazil. BM02 was identified by sequencing the V1–V2 regions of amplicon 16S rRNA. Total DNA was extracted using Kit ZR Bacterial DNA MiniPrep™ (D6005), and the integrity and quality of DNA were verified by electrophoresis. DNA was quantified using dsDNA HS Assay Kit (Invitrogen™, EUA). The primers used for sequencing were: 8F (5′ AGA GTT TGA TCC TGG CTC AG 3′), 519F (5′ GAG CAG CCG CGG TAA TAC 3′), 1492R (5′ GGT TAC CTT GTT ACG ACT T 3′), 519R (5′ GTA TTA CCG CGG CTG CTG 3′), and 968R (5′ GTA AGG TTC TTC GCG TT 3′). The obtained sequence was compared with GenBank sequences (NCBI), using Geneious (version 4.8.3; RRID: SCR_010519) software. For phylogenetic comparison, 15 NCBI sequences closest to BM02 were retrieved (Table S1), aligned (MAFFT v7, G-INS-i)^12^, and trimmed (TrimAl v1.3, Gappyout)^13^. The treated sequences were compared for maximum likelihood, through workflow RAxML-HPC2, in XSEDE (Cypres: https://www.phylo.org), model GTRGAMMA, and 1000 permutations^14^. *Pseudomonas aeruginosa* DSM 50,071 was chosen for rooting the phylogenetic tree since it is a species close to BM02 by the alignment test via Clustal Omega (Table S1). The 16S rRNA sequence of BM02 was deposited in NCBI (GenBank: OP410927.1).

#### Factorial design

2^3^-factorial design with a central compound was used, considering the production yield of BS (g/L) as a response variable; the independent variables were: pH (5–9), temperature (25–45 °C), and glycerol concentration (1–3%). The center point was performed in duplicate. The BM02 inoculum (1.5 × 10^8^ bacteria/mL; 5 mL) was transferred into an Erlenmeyer flask containing (100 mL) sterile mineral saline medium (MSM), with the following composition (g/L): K_2_HPO_4_, 4.0; Na_2_HPO_4_, 1.5; NaNO_3_, 1.0; MgSO_4_·7H_2_O, 0.2; CaCl_2_·2H_2_O, 0.02; FeCl_3_·6H_2_O, 0.02. The Erlenmeyer flasks were incubated at 180 rpm for five days to determine BS production and assess cell growth and emulsification production.

#### Cell growth and emulsification production

During the incubation period, the bacterial growth was spectrophotometrically monitored (Bel UV-M51; Ab _600 nm_). Aliquots were taken at time zero and every 24 h of incubation, and centrifuged (4000 rpm, 4 °C, 20 min) to obtain a cell-free broth. The emulsification index (IE24) was performed in tubes containing 2 mL of cell-free broth and 2 mL of mineral oil (Nujol). This mixture was vortexed at a high speed for two minutes and kept at rest for 24 h at 25 °C. The IE24 was calculated using the following formula: IE24 (%) = x/y × 100, where x and y represent the heights of the emulsified layer and the total layer of mixture, respectively^15^. The negative control was made with uninoculated MSM, and the positive control, with 1% sodium dodecyl sulfate (SDS).

#### BS quantification

After the incubation period, the microbial culture was centrifuged (4000 rpm, 4 °C, 20 min) and the cell-free broth was acidified to pH 2.0 using hydrochloric acid solution (6 N) and then kept at 4 °C overnight. The precipitate was centrifuged (4000 rpm, 4 °C, 10 min), resuspended with chloroform-ethanol (2:1, v/v) solution, vigorously mixed, and then kept at rest (24 h) in a phase separation flask. This procedure was repeated twice more. Afterward, the organic phase was evaporated in a rotary evaporator (Quimis, Brazil) to obtain crude BS, followed by drying in a circulated air oven to constant weight^16^.

### BS characterization

#### Stability analysis

Crude BS was dissolved in ultra-pure water (1 g/L) and submitted to analysis under the following conditions: temperature (10–100 °C), pH (3–11), and NaCl concentration (2–10%). The surface tension was assessed using a tensiometer (DataPhysics, Oca15 plus, Germany), by the hanging drop method, using an automatic video imaging system and program Oca 10/Oca 20.

#### Structural determination

The chemical structure of BS was determined by Fourier-Transform Infrared Spectroscopy (FT-IR), Nuclear Magnetic Resonance (NMR), and Electrospray Ionization Mass Spectrometry (ESI–MS). The FT-IR (Bruker FT-IR spectrometer, model Vertex 70, Germany) spectra was recorded within the region 4000–400 cm^−1^ and analyzed using the OriginPro 9.0 program. One-dimensional NMR analysis was performed on a Bruker Spectrometer (Model Avance III 400 MHz, Germany), operating at 400 MHz for ^1^H with a 5 mm probe at 25 °C. The experiment was carried out using the standard pulse programs of the NMR equipment and the spectral analysis was performed using the program MestreNova-6.0. The sample for NMR analysis was prepared from the crude BS at 12.5 mg/mL in CDCl_3_ and the chemical shifts were reported in ppm, with the solvent peak as a standard. ESI–MS analysis was performed on a QTOF spectrometer: quadrupole and TOF system for ultra-high resolution and mass precision (Bruker Daltonics, Model Compact, Germany) with direct injection. The mass spectrum was obtained in positive ion mode and the full scan data ranged from 50 to 1500 m/z. The equipment conditions were: capillary voltage of 3.8 kV, gaseous phase of nitrogen, gas flow of 4.0 L/min, gas temperature of 200 °C, and ion energy of 5.0 eV. The sample for ESI–MS analysis was prepared from the crude BS, dissolved in MS grade methanol at 0.01 mg/mL.

### BS toxicity

BS cytotoxicity was initially assessed after 72 h of exposure in different cell lineages (ATCC^®^): Vero (CCL-81, kidney of monkey; RRID: CVCL_0059), L-929 (CCL-1, fibroblast cloned from L strain; RRID: CVCL_IW05), MCF-7 (HTB-22™, metastatic breast adenocarcinoma; RRID: CVCL_1D39), and HEp-2 (CCL-23, human laryngeal carcinoma; RRID: CVCL_B7H7)^17^. Additionally, the BS cytotoxicity (12.5–500 µg/mL) was assessed at different times (1, 15, 30 and 60 min) after exposure to Vero CCL-81 cells (1.0 × 10^4^ cells/well), cultured (37 °C, 5% CO_2_) with Dulbecco’s Modified Eagle medium (DMEM, Gibco, USA), supplemented with 10% fetal bovine serum (FBS, Gibco, USA), penicillin (100 IU/mL), streptomycin (100 µg/mL) and 0.24% sodium bicarbonate (Sigma-Aldrich, USA)^18^. Untreated cells were kept with ‘DMEM’ as cell control (CC) and were able to promote reduction of MTT (Sigma-Aldrich, USA). The cell viability was calculated from the formula: Cell Viability (%) = [(AbBS_570nm_–AbBS_690nm_)/(AbCC_570nm_–AbCC_690nm_) × 100], where AbBS and AbCC, refer to the cells treated with BS and CC, respectively. The BS concentration able to reduce 50% of cell viability (CC_50_) was determined through linear regression. The BS selectivity for tumor cells was determined through the selectivity index (SI), calculated by the ratio: CC_50_ in non-tumor cell lineage/CC_50_ in tumor cell lineage. BS concentrations with SI ≥ 2 were considered potentially selective for tumor cells^19^.

### Antitumor activity of BS

#### Cell morphology assay

The antitumor effect of BS was assessed in MCF-7 cells using the dyes fluorescein diacetate (FDA, 7.5 µg/mL), Hoeschst 33.342 (Ho, 4.0 µg/mL), and propidium iodide (PI, 1.0 µg/mL) (Sigma-Aldrich, USA) for analysis of apoptosis or necrosis, through the presence or absence of apoptotic bodies, respectively^20^. Cells were exposed to BS (1–10 µg/mL) for 72 h with subsequent staining with fluorochromes and counting 300 cells/treatment. Phosphate buffered saline and Docetaxel (DT 50 µM) were used as negative (NC) and positive (PC) controls, respectively. The cells were analyzed by microscopy (Olympus BX 43, Olympus Microscopy, Europe) to assess the percentage of viable cells (FDA+/Ho+); in early (FDA+/Ho+), late (FDA+/Ho+/PI+) apoptosis; and necrosis (FDA−/Ho−/PI+)^21^.

#### Lactate dehydrogenase (LDH) release assay

LDH release by MCF-7 cells lineage was assessed using kit PierceTM LDH (Thermo Scientific, EUA). After the formation of the monolayer (1.0 × 10^4^/well), the cells were treated with BS (10–100 µg/mL) and incubated (37 °C, 5% CO_2_) for 72 h. LDH concentration was determined as: [A(BS)/A(control)], where: A (value obtained for each experimental group) = [(117,216 × Ab_490nm_)–(80,586 × Ab_680nm_)]^22^. The PC, provided by the kit, represented 100% membrane lysis. Standard chemotherapy (DT50: docetaxel, 50 µM) was applied.

#### Clonogenic assay (CL)

MCF-7 cells were seeded in a 24-well plate (5 × 10^4^ cells/well). After 24 h, treatments with BS (1–10 µg/mL), NC (phosphate buffered saline), and PC (DT50, 50 µM) were added to the cultures for 72 h. Then, the cells were trypsinized and counted, and 250 viable cells were seeded in 6-well plates, kept immobile for 14 days. For staining, the plates were washed with NC and fixative solution (ethanol/acetone, 1:2, v/v) for 10 min, followed by staining with 5% Giemsa (Dinamica, Brazil). Then, the plates were washed with distilled water and photographed, and the colonies were counted using the software Zen 2.3 (Zeiss, Germany). The survival fraction of the treated cells was obtained in relation to the NC (100% survival fraction)^23^.

### Antiviral activity of BS

The BS effect was assessed against enveloped (Herpes Simplex Virus Type 1: HSV-1, KOS strain; Murine Coronavirus Type 3: MHV-3; Respiratory Syncytial Virus A: RSV, strain Long), and non-enveloped (Poliovirus: PV-1, strain Sabin) viruses, kept in a biobank at − 80 °C, in the Virology Laboratory of State University of Londrina, Londrina-PR, Brazil. Previously, the viral stocks were obtained by inoculating Vero (HSV-1 and PV-1), L-929 (MHV-3), or HEp-2 (RSV) cells, grown in culture flasks 25 cm^2^. The cells were monitored daily for 5 to 10 days to observe the cytopathic effect (CPE). Viral titration was carried out using the TCDI 50 technique^24^. Then, the viruses were incubated (1, 15, 30 and 60 min) with BS (500–6.25 µg/mL). Afterward, an aliquot of each suspension was diluted 10 or 100 times for inoculation in the respective cells (MOI 0.1), grown in 96-well microplates (10^4^ cells/mL), and at the non-cytotoxic dose. The plates were incubated (37 °C, 5% CO_2_) with daily observation of the presence or absence of CPE, for 72 h. The virus control (VC; infected and untreated cells) was performed simultaneously and under the same conditions, but without BS. The antiviral activity was determined by the reduction in viral titer concerning VC^25^.

### Antimicrobial activity of BS

It was assessed by the microdilution method according to the recommendations of the National Committee for Clinical Laboratory Standards, against *Staphylococcus aureus* (ATCC 25,923), *Escherichia coli* (ATCC 25,922), *Enterococcus faecium* (ATCC 6569) and *Salmonella choleraesuis* (ATCC 10,708), and the strains *Pseudomonas aeruginosa* (INCQS 2742) and *Candida albicans* (INCQS 72,000), provided by the National Institute for Quality Control in Health, Fundação Oswaldo Cruz, Brazil. In 96-well flat-bottom microplates, serial dilutions of BS (50,000–25 µg/mL) were made in sterile Mueller–Hinton broth. Subsequently, a suspension of each microorganism (1.5 × 10^8^ microorganism/mL) was inoculated in the exponential growth phase, and incubated at 37 °C for 24 h for bacteria and 48 h for yeast. The minimum inhibitory concentration (MIC) was determined by adding 4 µL of developing solution (2,3,5 triphenyl tetrazolium chloride, 1%; Sigma Chem. Co., USA). The lowest concentration that showed no color in the wells was established as MIC. Tetracycline (Prati-Donaduzzi, Brazil) and fluconazole (Cimed, Brazil) were used as PC for bacteria and yeast, respectively.

### Statistical analysis

The statistical analyses of the factorial design were made using the program Statistics for Windows, version 10.0 by Stat Soft (1984–2011), generating the ANOVA table and Pareto and response surface charts. The descriptive analyses of the BS stability tests were made using Microsoft Excel, version 2010. For the analysis of the cytotoxic, antitumor, and antiviral activities, One-Way variance analysis (ANOVA) was used, followed by Tukey’s test (p ≤ 0.05), using the software GraphPad Prism^®^, version 7.00 (RRID: SCR_002798). Sample normality was assessed by the Shapiro–Wilk test.

## Results

Alignment test analyses by Clustal Omega indicate 100% similarity of the BM02 sequence with other 16S rRNA sequences from *Pseudomonas* spp., including *P. aeruginosa* (Table S1). These analyses also suggest that all 16S rRNA sequences recovered from NCBI are close, indicating very recent divergence, with the BM02 showing high similarity (> 97%) with other *Pseudomonas* sequences. According to the phylogenetic analyzes, the BM02 sequence is closer to the *P. aeruginosa* DSM 50,071 and NBRC 12,689 sequences (Fig. S1). *Pseudomonas aeruginosa* NBRC 12,689 was determined for the rooting of the phylogenetic tree due to the close proximity of the BM02 obtained in the test of alignment by Clustal Omega (Fig. S1).

Factorial design analyses reveal variation in the BS production concerning the variables temperature (p = 0.0317) and glycerol content (p = 0.0408); only the pH variable was not significant (p = 0.0651) (Table S2). As described in Table 1, the factorial combinations of test 1 (pH 5.0, 25 °C, and 1.0% glycerol) promoted the highest yield in BS production (2.28 mg/mL), although the central point replicates (tests 9 and 10) also stands out in the BS production, with the average of the central point equivalent to 82% of the highest obtained value.

**Table 1:** Factorial combinations for biosurfactant (BS) production by *Pseudomonas aeruginosa* BM02.

Test	pH	Temperature (°C)	Glycerol concentration (%)	BS yield (mg/mL)
1	5.0	25	1.0	2.28
2	5.0	45	1.0	0.70
3	9.0	25	1.0	0.96
4	9.0	45	1.0	0.54
5	5.0	25	3.0	1.91
6	5.0	45	3.0	1.11
7	9.0	25	3.0	1.47
8	9.0	45	3.0	1.86
9	7.0	35	2.0	1.91
10	7.0	35	2.0	1.85

**Figure 1:**
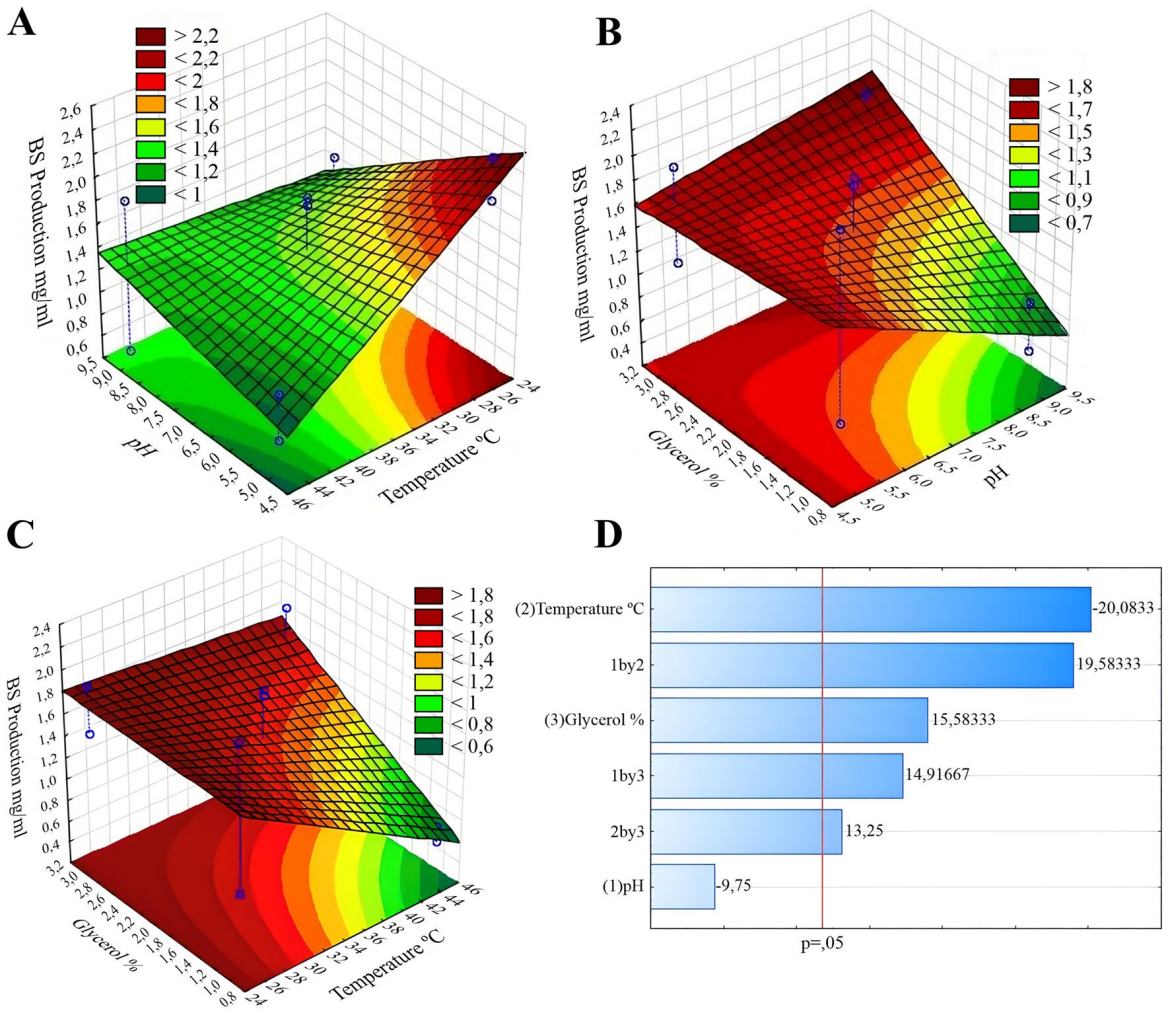
Factorial Design Response Surfaces: (**A**) pH vs temperature; (**B**) pH vs glycerol content; (**C**) temperature vs glycerol content; (**D**) Significant variables from the Pareto chart.

Pareto chart (Fig. 1D) analyzes reveal that the pH variation in the range studied did not influence the BS production. In turn, the temperature influenced the BS production (Fig. 1A, C), being the 25 °C was the most favored for BS production. The pH was not a significant variable alone, but the interactions of pH 5.0 and temperature of 25 °C (Fig. 1A), and pH 9.0 and 3.0% glycerol (Fig. 1B) contributed to the higher BS yield.

**Figure 2:**
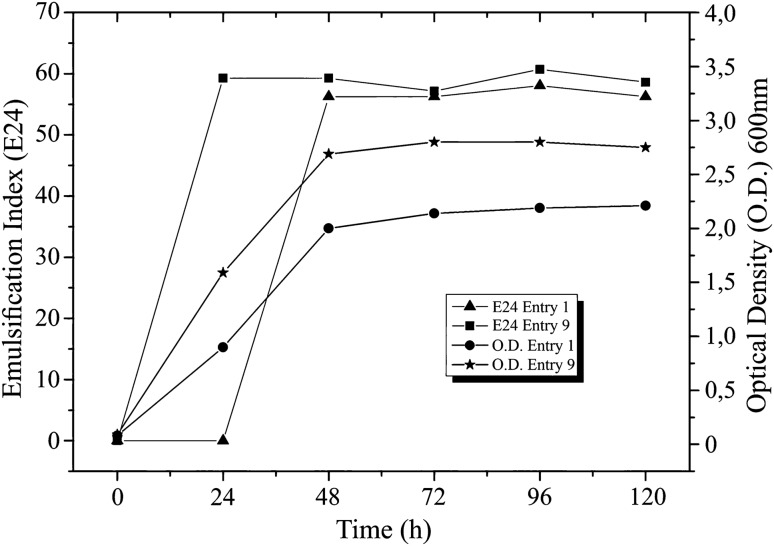
Emulsification index and growth of *Pseudomonas aeruginosa* BM02.

For purposes of follow-up of BS production by *P. aeruginosa* BM02, the IE24 and the optical density of tests (entry) 1 and 9 (tests with higher yield based on factorial design) were monitored. The IE24 resulted within the range of 54% to 60% (Fig. 2), SDS was 72% and uninoculated MSM did not form emulsified. The optical density (OD) varied from 0.70 to 2.0 in the exponential growth phase, between 24 and 48 h (Fig. 2).

The BS demonstrated stability in all tested conditions (Table 2), with the minimum value of surface tension (~ 27 mN/m) at pH 3.0, 2% NaCl and 80 °C. The assay control (uninoculated MSM) presented a surface tension ranging from 54.2 to 67.8 mN/m, according to the glycerol concentration (1% to 3%), and ultrapure water was of 72.45 mN/m.

**Table 2:** Stability of the biosurfactant by *Pseudomonas aeruginosa* BM02.

Variable		Surface tension (mN/m)	Variable		Surface tension (mN/m)	Variable		Surface tension (mN/m)
pH	3	27.95 ± 0.4	NaCl (%)	2	27.29 ± 0.5	Temperature (°C)	10	27.41 ± 0.1
	5	28.35 ± 0.4		4	28.01 ± 0.2		30	27.67 ± 0.0
	7	33.22 ± 1.6		6	31.80 ± 0.4		55	27.44 ± 0.3
	9	32.70 ± 0.6		8	31.39 ± 0.2		80	26.83 ± 0.3
	11	33.81 ± 0.6		10	31.44 ± 0.2		100	29.26 ± 0.3

Results of thre replicates are expressed as mean ± SD.

According to FT-IR analyses, the important absorption bands observed in BS were located at 3318, 2923, 2855, 1736, and 1036 cm^−1^ (Fig. S2). The broad absorption band at 3318 cm^−1^ is characteristic of hydroxyl group stretching vibrations. The bands at 2923 and 2855 cm^−1^ refer to the asymmetric and symmetric vibrations of the stretching of C–H bonds of *sp*^3^ carbon, respectively. These signals may come from the long aliphatic chains of the RL. The peak with maximum absorption (1736 cm^−1^) is characteristic of the stretching vibration of the ester C=O bond, superimposed with the stretching band of the carboxylic acid carbonyl group (1707 cm^−1^). The absorption bands of 1300–1000 cm^−1^ are characteristic of stretching vibrations of C–O bonds, indicating the presence of alcohol and ester functional groups. The most intense band in this region at 1036 cm^−1^ corresponds to the stretching vibration of the –C–O–C– group of the cyclic structure of the rhamnose rings. These results show strong evidence that the found signals are consistent with the functional groups of the RL.

The ^1^H NMR spectrometry (Fig. S3) analysis revealed signals with chemical shifts (δH) between 0.86 and 0.89 ppm, which are characteristic of the methyl groups in both the rhamnose moiety and the fatty acid. The signals between δH 1.36 to 2.63 ppm refer to the fatty acid chain hydrogens, and the rhamnose moiety hydrogens appear between δH 3.29 and 4.88 ppm. In addition, the three signals in the range of δH 4.85 to 5.42 ppm stand out, which show two rhamnose units in the structure and two bonded fatty acids, since the shift at 5.42 ppm is characteristic of CH that connects the fatty acids. There was no clear evidence of the presence of unsaturated fatty acids, as the spectrum did not show relevant signals with δH > 8.0 ppm. Thus, based on the attributions made by the FT-IR and NMR analyses, it can be stated that the BS consists of RL. The low resolution of the NMR spectrum can be an indication that the BS contemplates a mixture of RL congeners.

BS was submitted to mass spectrometry analysis to identify its lipid side chains and the structural homologs (Fig. S4). Table 3 shows the putative composition of the RL congeners of BS, consisting of a mixture of mono-RL (one rhamnose unit) and di-RL (two rhamnose units). Due to the analysis in positive mode, one RL was detected in the H^+^ form, while the others were mainly detected with the addition of Na^+^ counter-ions. The obtained BS might be a complex mixture of these RL, emphasizing the mono- and di-RL containing two saturated C_10_ fatty acids.

**Table 3:** Putative chemical composition of biosurfactant by *Pseudomonas aeruginosa* BM02.

Rhamnolipids	[M]	[M + H]^+^ *m/z*	[M + Na]^+^ *m/z*	[M + K]^+^ *m/z*
Mono-rhamnolipids				
Rha-Fa-C_8:2_	302		325	
Rha-Fa-C_10:2_	330		353	369
Rha-Fa-C_12:2_	358		381	
Rha-Fa-C_8_-Fa-C_10_ or Rha-Fa-C_10_-Fa-C_8_	476		499	
Rha-Fa-C_10_-Fa-C_10_	504		527	
Rha-Fa-C_10_-Fa-C_12:1_ or Rha-Fa-C_12_-Fa-C_10:1_ or Rha-Fa-C_10_:1-Fa-C_12_ or Rha-Fa-C_12:1_-Fa-C_10_	530		553	
Di-rhamnolipids				
Rha-Rha-Fa-C_10:1_-Fa-C_10:1_ or Rha-Rha-Fa-C_10_-Fa-C_10:2_ or Rha-Rha-Fa-C_10:2_-Fa-C_10_	646	647		
Rha-Rha-Fa-C_10_-Fa-C_10_	650		673	

Rha, rhamnose; Fa, fatty acid; C8:x to C14:x: length of the fatty acids and presence of unsaturations.

**Figure 3:**
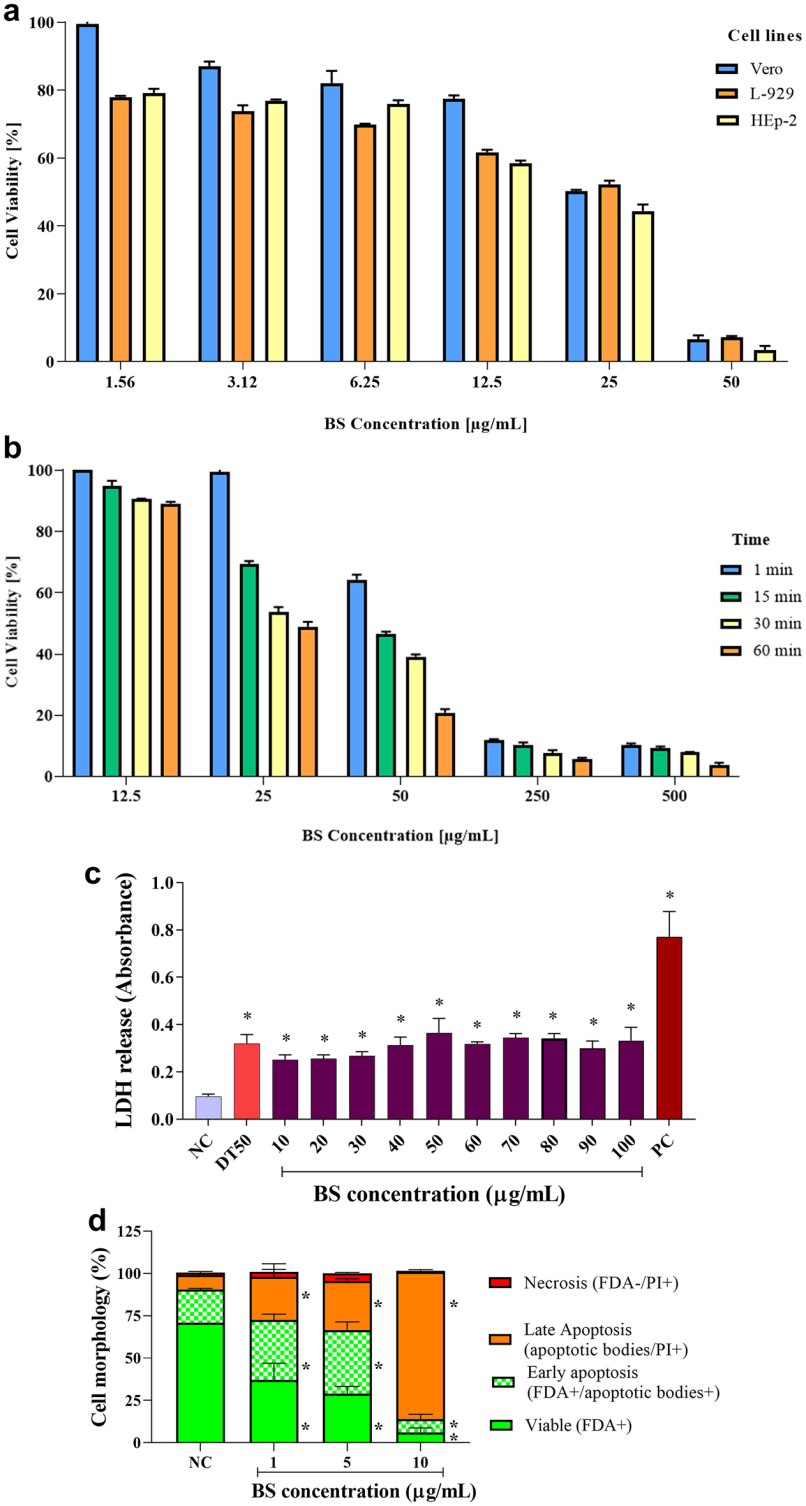
Percentage of cell viability (**A**) after 72 h of exposure in Vero, L-929 and Hep-2 cells and (**B**) after exposure at different times (1–60 min) in Vero cells; and antitumor activity by (**C**) lactate dehydrogenase (LDH) release and (**D**) assessment of cell morphology of biosurfactant and antitumor activity by (**C**) lactate dehydrogenase (LDH) release and (**D**) assessment of cell morphology (MCF-7) of biosurfactant (BS) from *Pseudomonas aeruginosa* BM02.

The cytotoxicity of BS on Vero, L-929 and HEp-2 cells were dose-dependent, with a reduction in viability proportional to the increase in concentrations, after 72 h of exposure (Fig. 3A), with CC_50_ of 25 µg/mL, 27.25 µg/mL, and 20.16 µg/mL, respectively. Due to their well-established use in viral replication studies, Vero cells were used in determination trials of the antiviral and antitumor potential of BS. The BS concentration of 12.5 µg/mL kept the cells viable (> 88.8%) at all assessed times (Fig. 3B). A similar result was obtained only for the two shortest times (1 and 15 min) at 25 µg/mL, reducing the viability to 54.9% and 50% at 30 and 60 min of exposure, respectively. At 50 µg/mL, Vero cells maintained 63.2% cell viability in 1 min, reducing to less than 50% in the other assessed times. However, lower viability (CC_50_ = 5.71 µg/mL) was found for MCF-7 cells after 72 h of exposure, and the LDH release assay was performed to assess whether BS alters plasma membrane integrity (Fig. 3C). All tested BS concentrations decreased MCF-7 cell viability (CC_50_ = 4.43 µg/mL) to levels close to the standard chemotherapy (DT50). The SI of BS found for MCF-7 was 4.4, considered potentially selective for this tumor cell lineage. The antitumor activity of BS in MCF-7 was assessed (Fig. 3D), and after 72 h of treatment, the number of viable cells reduced by 37%, 29%, and 6%, at BS concentrations of 1.0, 5.0, and 10 µg/mL, respectively. Furthermore, BS induced cell death observed by the increase of cells in initial apoptosis, 35% and 37% at concentrations of 1.0 and 5.0 µg/mL, respectively; similar results were observed to the positive control of the assay (36.5%; data not shown). At the highest BS concentration (10 µg/mL), most cells (87%) were in terminal apoptosis. BS appears not to induce necrosis in MCF-7 cells, or weakly stimulate this cellular event, under assay conditions.

**Figure 4:**
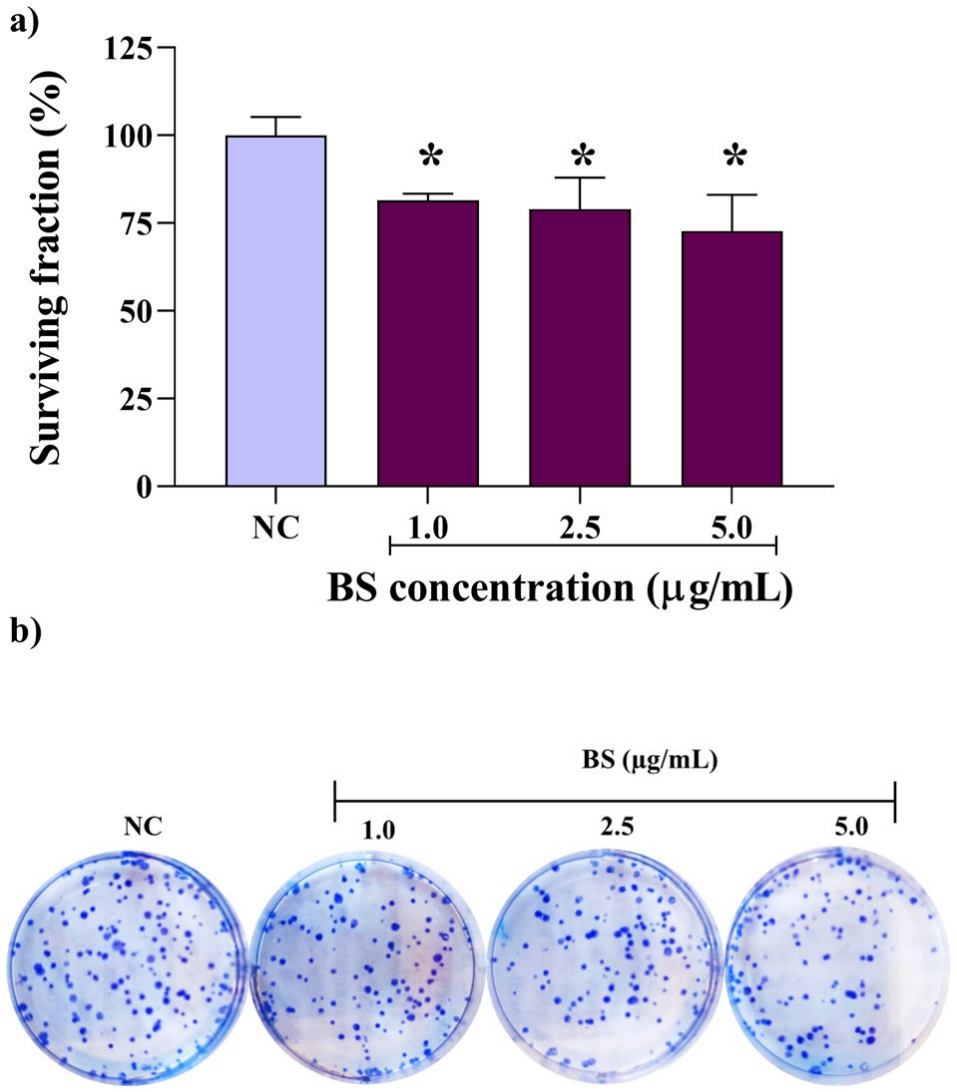
Surviving MCF-7 cells (%) after treatment with biosurfactant (BS) by *Pseudomonas aeruginosa* BM02: (**A**) proliferative capacity of BS, expressed in percentage (%); (**B**) formation of colonies.

From the clonogenic assay analyses, it was identified that MCF-7 cells treated with BS equivalent and lower concentrations of CC_50_ (1.0, 2.5 and 5.0 µg/mL) reduced the cell viability to 81.5%, 79.0%, and 72.8%, respectively (Fig. 4A). Furthermore, BS decreased the ability of MCF-7 cells to form colonies (Fig. 4B). DT (50 µM) completely inhibited colony formation.

**Figure 5:**
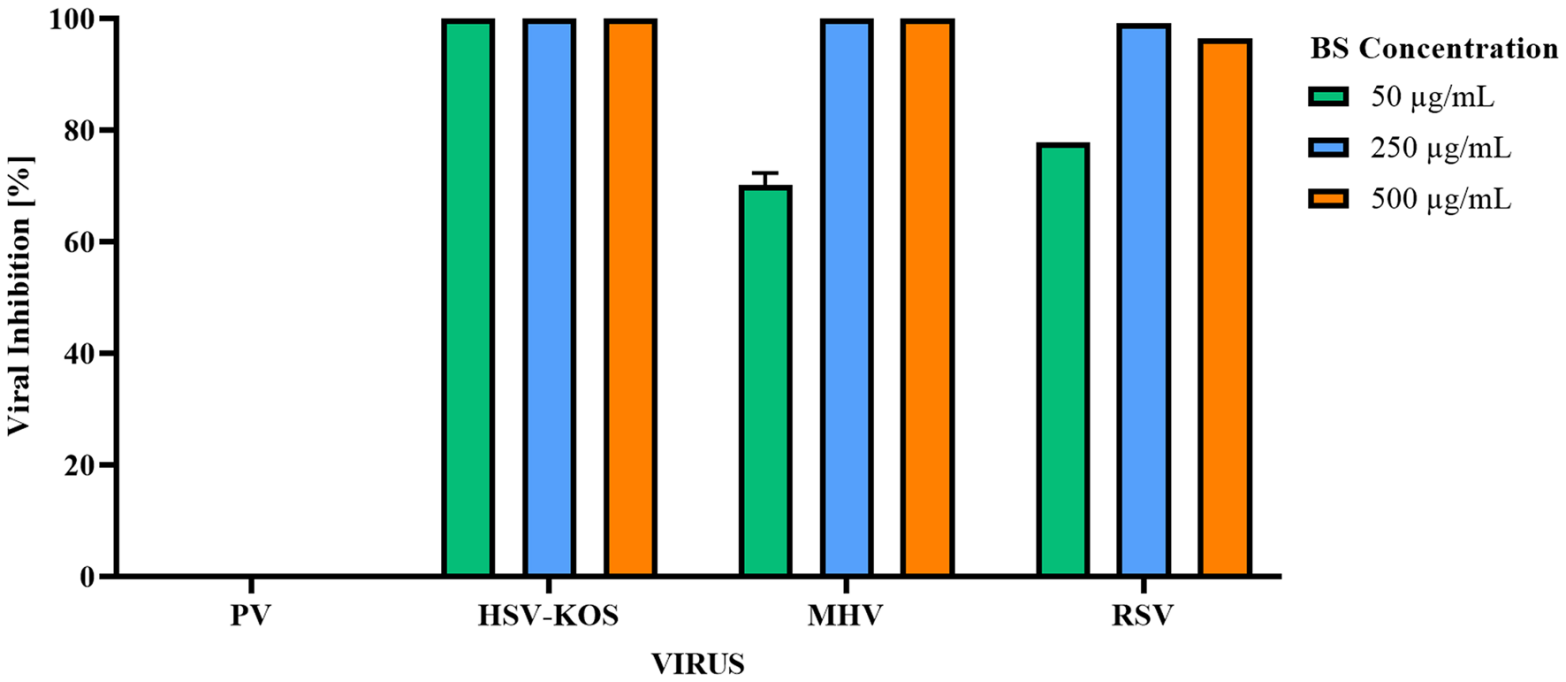
Virucidal effect (percentage of inhibition) of biosurfactant (BS) by *Pseudomonas aeruginosa* BM02 after 1 min of exposure.

Analyses of the virucidal effects of BS highlight its ability to inhibit 96.4% of the CPE of all enveloped viruses (HSV-1, MHV-3 and RSV) evaluated, in 1 min contact, at 250 µg/mL (Fig. 5). At 50 µg/mL and same contact time, the absence of CPE (100%) was observed only for HSV-1, with a reduction to 71.6% and 77.8% for MHV-3 and RSV, respectively. In the other contact times (15, 30, and 60 min), the inhibition occurred for all enveloped viruses, from the BS concentration of 50 µg/mL. Based on BS virucidal effect results, the anti-HSV-1 activity of BS was also investigated, showing total CPE inhibition after 1 min of exposure, up to 12.5 µg/mL, without cytotoxicity. At BS concentration of 6.25 µg/mL, the absence of CPE depended on the exposure time, with 85% inhibition in 30 and 60 min, reduction to 68% in 15 min, and no inactivation when exposed to 1 min of contact. However, BS did not inactivate the Poliovirus (PV-1), a non-enveloped virus, at all tested conditions.

The antimicrobial potential of BS was also investigated, and the analyses suggest that Gram-positive bacteria (*S. aureus* and *E. faecium*) were more susceptible to BS. In contrast, Gram-negative bacteria (*S. choleraesuis*, *E. coli* and *P. aeruginosa*) and yeast (*C. albicans*) showed weak antimicrobial activity (Table 4).

**Table 4:** Minimum inhibitory concentration (MIC, µg/mL) of the biosurfactant produced by *P. aeruginosa* BM02 against pathogenic microbial strains.

Microbial Strains	Biosurfactant	Tetracycline	Fluconazole
*Staphylococcus aureus*	50	25	NT
*Escherichia coli*	NI	25	NT
*Enterococcus faecium*	50	25	NT
*Salmonella choleraesuis*	50,000	25	NT
*Pseudomonas aeruginosa*	NI	50	NT
*Pseudomonas aeruginosa*	50,000	NT	50

Tetracycline and fluconazole were used as positive controls for bacterial and fungal strains, respectively.

The tested compounds were added at different concentrations (50,000–25 µg/mL) to the microbial strains for 24 h for bacteria and 48 h for yeast. MIC was determined as the lowest concentration, which did not show any color. NI: There was no inhibition up to the highest tested concentration (50,000 µg/mL). NT: Not tested.

## Discussion

*Pseudomonas aeruginosa* is a common, Gram-negative environmental organism, known to exhibiting adaptable metabolism and ability to colonize a range of niches^26^. An important characteristic of *P. aeruginosa,* reported in the literature, is its ability to produce BS. However, its function for bacterial cells is still not fully understood^27^; some physiological applications have been suggested, such as solubilization of insoluble substrates, adhesion to interfaces in natural ecosystems, and metal complexation, as a form of adaptation of microorganisms in metal-rich environments, to reduce or inhibit cellular toxicity^28^. Bonds between metal and BS micelles can occur by attraction between anionic BS and non-ionic metallic forms, or by the formation of chelates on the surface of micelles^1^. In this work, the BM02 lineage was isolated from the soil of the bauxite mining industry, an aluminum-rich environment that may have influenced the BS production.

Factorial design analyses revealed the production of BS by *P. aeruginosa* is influenced by pH and glycerol concentration, corroborating preliminary studies^29,30^, namely: moderately acidic pH (~ 6.0), and glycerol concentration between 0.5% and 3.0% are considered optimal. However, unlike our findings, Silva et al.^30^ did not record differences in BS production by *P. aeruginosa* at temperatures up to 37 °C. These authors provided no information on the biological origin of the bacterial strain. In our study, the BM02 strain was isolated from the acid soil of a Brazilian municipality with temperatures around 28 ± 6 °C^31^, a temperature range close to that considered optimal for BS production, according to factorial design analyses. These conditions may have favored the best BS yield, under the tested culture conditions. The carbon source in the culture medium is an essential element in the BS production^7^. However, it is essential to establish sustainable production routes, employing environmentally friendly bioprocesses and reagents, and glycerol is a highly available and low-cost option applied to green chemistry^32^. Glycerol was used as the only carbon source in this work, an alternative substrate commonly used in the production of microbial BS. The use of factorial design, allied to the use of industrial process wastes, is an important tool to optimize BS production conditions from independent variables, enabling the prediction of adequate parameters to achieve the best BS yield and correct disposal of glycerol residues^33^. However, the yield of BS produced by *P. aeruginosa* BM02 was considered low when compared to other studies, making it necessary to evaluate the production capacity of the bacteria species against different growth and synthesis factors.

The obtained BS showed the highest IE24 (60%) using 2% glycerol. The emulsified production remaining constant throughout the monitored period (120 h). Emulsion production is a widely used technique to determine the presence of BS in cell-free broth^34^, considering a satisfactory emulsifying agent when forming 50% emulsion, maintained for 24 h^35^. Emulsification activity is an important characteristic used to evaluate the potential applicability of BS in industry (e.g., medicine, cosmetic, bioremediation)^4^, and the presented results strengthen the potential of the obtained BS for industrial application.

The application of BS depends on its ability to remain stable under different conditions, considering variations in pH, temperature, and salinity^36^. Joshi et al.^37^ reported that the BS was stable at a temperature of 80 °C, and variations in pH and NaCl between 6 and 12, and 1–7%, respectively; however, it increased its surface tension at NaCl concentrations greater than 10%, and at acidic pH, with precipitate formation. Gudiña et al.^36^ observed that the surface activity of BS remained stable at temperatures from 25 to 60 °C, however, there was lower surface tension at pH 7, and the highest at pH below 6, showing turbidity and partial precipitation of BS. Giri et al.^38^ showed minimum values of surface tension at pH 7 and 20 °C, with reduction of BS activity at temperatures higher than 100 °C. In this work, BS showed stability in all tested variables; there was no formation of precipitate or reduction of tensioactive activity. Some studies characterize the BS as primary metabolites associated with exponential bacterial growth^39^. In this work, it was not possible to determine in which phase of bacterial growth the BS was produced, enabling the performance of studies in this theme.

According to FT-IR and ^1^H NMR analyses, the found signals are consistent with the functional groups of the RL described in the literature^3,27^. In this study, the putative composition of the RL congeners of BS was described as consisting of a mixture of mono- and di-RL. The most abundant *P. aeruginosa* RL reported in the literature contains fatty acids from C_8_ to C_14_, considering that C_10_ is the most common^40^. Furthermore, based on the Table 3 data, it can be observed that the peaks at *m/z* 499, 553, and 647 provide the possibility of RL congeners with different dispositions of the fatty acid chains. It should be pointed out that there was no quantification of each congener; therefore, some of them, especially the less common ones, may be present in minor quantities. This may explain the fact that the presence of unsaturated chain congeners was not evidenced by NMR analysis, despite the very weak signals in the 7.0–8.2 ppm region, but was detected in mass spectrometry. Finally, the information provided by mass spectrometry revealed that there may be both mono- and di-rhamnose moieties, as well as mono- and di-lipid congeners. In addition, high diversity was observed in the length and the nature (saturated or unsaturated) of the lipid chains in relation to the most common chemical structures for the species.

The cytotoxic activity of BS was determined by the MTT assay, which is widely used to assess cellular metabolic activity^41^, evidencing selective toxicity for MCF-7 cells (CC_50_ = 5.71 µg/mL), a breast cancer cell line, corroborating with studies on antitumor/anticancer potential of BS vary according to cell lines and exposure time^42,43^. Similar results were obtained by RL by *P. aeruginosa* BN10, with CC_50_ = 8.6 µg/mL for MCF-7^44^. RL by *P. aeruginosa* B189 showed CC_50_ = 6.25 µg/mL for MCF-7, but was not cytotoxic (CC_50_ > 50 µg/mL) for Vero cells, and lung (NCI-H187), and oral epidermoid (KB) cell carcinomas^45^. The LDH release assay analyses suggest the cytotoxic safety of the obtained BS; all tested BS concentrations decreased MCF-7 cell viability (SI = 4.4) to levels close to the standard chemotherapy (DT50), similar to results reported by Tambone et al.^46^ for RL by *P. aeruginosa*. The antitumor activity of RL-type BS was demonstrated for different cells^47,48^, strengthening the feasibility of indicating the obtained BS as an alternative to conventional chemotherapeutic agents.

In addition to compromising the membrane integrity, BS induced cell death by apoptosis and decreased the clonogenic capacity of MCF-7 cells, probably due to the amphiphilic nature of the molecule, similar to that reported in other studies^49^. Cytotoxic or cytostatic activity of BS seems to depend on interaction/penetration into the lipid bilayer influenced by the chain size and the micelle formation capacity^50^. However, the greater cytotoxicity found for MCF-7 cells does not rule out the hypothesis that our BS may interfere with other mechanisms, resulting in cancer cell apoptosis. The potentially selective and effective inhibition of MCF-7 cell proliferation, at low BS concentrations, are promising for anticancer therapies, considering the limited efficacy of most available therapeutic drugs.

BS showed virucidal effects against enveloped viruses HSV-1, MHV-3 and RSV after 1 min contact. The anti-HSV effect of BS has been widely investigated^51^; however, the activity for RSV virus is still little reported. The inactivation of RSV shows promise in the investigation of BS antiviral properties in general therapy for respiratory viruses, where the use of chemical surfactants is indicated^52^. The virucidal effect of BS is attributed mainly to its physicochemical characteristics that favor interaction with the viral envelope lipid membrane. Many viruses of epidemiological importance have this structure, which is fundamental for the recognition of the host cell and the onset of the infectious cycle^51^. Molecules containing fatty acids are strongly suggested as inactivating enveloped viruses^53,54^, especially HSV-1, the most susceptible viral strain to BS. In the present study, BS presented RL containing units of C_10_ saturated fatty acids in its composition, capable of interact physiochemically with the viruses, which may explain the viral inactivation. Additionally, several BS were assessed against coronaviruses, using SARS-CoV-2 or similar species^55^. In this study, the murine coronavirus (MHV-3) was used as a model for assessment the virucidal effect of BS, as it belongs to the same genus of SARS-CoV-2^56^. The inactivation of MHV-3 after 1 min of contact makes BS a promise in the coronaviruses’ management, contributing to world public health. However, BS did not inactivate the Poliovirus (PV-1), a non-enveloped virus, at all tested conditions.

The BS also has antimicrobial activity against pathogenic bacteria and yeast. Reports on the antimicrobial activity of BS by *P. aeruginosa* against Gram-positive and Gram-negative bacteria and yeast investigated in this research, as well as other microorganisms of industrial and medical importance, have already been described in the literature^57,58^. Gram-positive bacteria usually are more susceptible to BS^59^, whose inhibition mechanism occurs through the insertion of fatty acid components (short acyl tails) into the bacterial cell membrane, causing a break between the cytoskeletal elements and the plasma membrane^57,58^. However, Gram-negative bacteria are normally more resistant to BS, possibly due to the presence of lipopolysaccharide and extracellular polymers in the outer membrane, which is little permeable to hydrophobic and amphipathic molecules^59^. The weak antifungal activity can be attributed to the BS composition, as suggested by Rodrigues et al.^60^; compared to di-RL, mono-RL congeners interact weakly with the fungal phospholipid bilayer, and in our heterogeneous mixture of rhamnolipids, the ratio of mono-RL is higher.

Surfactant activity due to the ability of their amphiphilic regions to interact with membrane structures, channel formation and significant conformational changes, has been proposed for biosurfactants^61^. In this study, we suggest that this non-specific mechanism is mainly responsible for the found biological activities. However, other mechanisms by which biosurfactants affect membrane integrity such as the property of adhesion to cell surfaces, ability to internalize the plasma membrane, rupture of the cytoskeleton, accumulation of intramembranous particles, increase in electrical conductance of the membrane, and induction of intracellular signaling pathways infections should not be ruled out^62,63^. New studies will contribute to elucidating the structure–activity relationships of this BS.

## Conclusions

The possibilities of enhancing BS production by *P. aeruginosa* BM02 using media composition design and resulting BS properties were studied. The BM02 lineage was isolated in the Brazilian Amazon region, under the influence of the bauxite extraction, and identified as *P. aeruginosa*, a BS-producing bacterium RL-type under cultivation conditions using a temperature of 25 °C, pH 5 and 1.0% glycerol. The applicability of prediction was verified for media composition optimization with significant BS yield above 2.0 mg/mL and stability of surfactant properties for up to 120 h. The applications of BS include strong virucidal effect against enveloped viruses and selective antitumor activity for breast tumor cells, as well as inhibition of the growth of Gram-positive bacteria. Our results indicate RL-type BS from *P. aeruginosa* BM02 as a promising compound for the development of new antitumor and antimicrobial prototypes, as a management strategy for important public health concerns. The Amazonian mining environment, particularly that of bauxite, is still little known in microbiological studies, but it may present itself as an important microbial reservoir with specific biological functions, as presented in this work. This study also enables discussions on the use of contaminated soil and mining waste, especially from the Brazilian Amazon region, the largest rainforest in the world, for the development of low-cost and ecologically friendly processes.

### Supplementary Information

Supplementary Information.

## Data Availability

The authors confirm that the data generated or analyzed during this research are available within the article. The 16S rRNA sequence of BM02 is available in National Center for Biotechnology Information (NCBI), with the accession number OP410927.1 (https://www.ncbi.nlm.nih.gov/nuccore/OP410927).
