# Supplementary material for: Production and characterization of rhamnolipids by Pseudomonas aeruginosa isolated in the Amazon region, and potential antiviral, antitumor, and antimicrobial activity
Source: Sci Rep. 2024 Mar 12;14:4629. doi: 10.1038/s41598-024-54828-w (PMC10933355; doi:10.1038/s41598-024-54828-w)

SUPPLEMENTARY MATERIAL

Production and characterization of rhamnolipids by *Pseudomonas aeruginosa* isolated in the Amazon region, and potential antiviral, antitumor, and antimicrobial activity

Sidnei Cerqueira dos Santos[a1*], Chayenna Araújo Torquato[a1], Darlisson de Alexandria Santos[b], Alexandre Orsato[c1], Karoline Leite[c1], Juliana Mara Serpeloni[c2], Roberta Losi-Guembarovski[c2], Erica Romão Pereira[c2], André Luiz Dyna[c3], Mario Gabriel Lopes Barboza[c3], Matheus Hideki Fernandes Arakawa[c3], José Augusto Pires Bitencourt[d], Sebastião da Cruz Silva[a2], Giulian César da Silva Sá [a1], Pamela Dias Rodrigues[e], Cristina Maria Quintella[e], Lígia Carla Faccin-Galhardi[c3]

[a] Federal University of Southern and Southeast Pará (Unifesspa), [1]Biology College, [2]Chemistry College, Marabá-PA, ZIP CODE 68500-000, Brazil

[b] Federal University of Pernambuco (UFPE), Department of Fundamental Chemistry, Recife-PE, ZIP CODE 50740-560, Brazil

[c] State University of Londrina (UEL), [1]Department of Chemistry, [2]Department of General Biology, [3]Department of Microbiology, Londrina-PR, ZIP CODE 86057-970, Brazil

[d] Vale Technological Institute (ITV), Belém-PA, ZIP CODE 66055-090, Brazil

[e] Federal University of Bahia (UFBA), Department of General and Inorganic Chemistry, Salvador-BA, ZIP CODE 40170-115, Brazil

[*] Corresponding author: sidnei.cerqueira@unifesspa.edu.br; +55 (94) 2101-7121

Table S1. Percentage of sequence identity of 16S rRNA from BM02 and *Pseudomonas* spp. retrieved from the NCBI. Alignment was generated by Clustal Omega v2.1.

	Accession Numbers	Names	1	2	3	4	5	6	7	8	9	10	11	12	13	14	15	16
1	NR 112062.1	*Pseudomonas resinovorans* ATCC 14235	100	96.92	96.64	97.21	97.40	97.30	97.30	97.16	97.16	97.10	97.16	97.41	97.41	97.41	97.28	97.34
2	NR 113646.1	*Pseudomonas alcaligenes* NBRC 14159	96.92	100	99.86	98.15	97.60	97.60	97.55	97.42	97.55	97.42	97.42	97.60	97.60	97.60	97.55	97.60
3	NR 114472.1	*Pseudomonas alcaligenes* ATCC 14909	96.64	99.86	100	97.94	97.58	97.39	97.36	97.22	97.36	97.23	97.23	97.39	97.39	97.39	97.49	97.39
4	NR 043289.1	*Pseudomonas otitidis* MCC10330	97.21	98.15	97.94	100	98.63	98.52	98.55	98.41	98.14	98.07	98.40	98.56	98.50	98.50	98.60	98.62
5	NR 113599.1	*Pseudomonas aeruginosa* NBRC 12689	97.40	97.60	97.58	98.63	100	100	100	99.86	100	99.86	99.86	100	100	100	100	100
6	NR 114471.1	*Pseudomonas aeruginosa* ATCC 10145	97.30	97.60	97.39	98.52	100	100	100	99.86	99.65	99.52	99.86	99.93	99.93	99.93	100	99.87
7	ON255834.1	*Pseudomonas sp.* 20TX0129	97.30	97.55	97.36	98.55	100	100	100	100	99.59	99.65	100	100	100	100	100.	99.93

	Accession Numbers	Names	1	2	3	4	5	6	7	8	9	10	11	12	13	14	15	16
8	ON255854.1	*Pseudomonas sp.* 20TX0150	97.16	97.42	97.22	98.41	99.86	99.86	100	100	99.65	99.66	100	99.86	99.86	99.86	100	99.79
9	ON255866.1	*Pseudomonas sp.* 20TX0168	97.16	97.55	97.36	98.14	100	99.65	99.59	99.65	100	99.72	100	99.59	99.59	99.59	100	99.65
10	ON255867.1	*Pseudomonas sp.* 20TX0170	97.10	97.42	97.23	98.07	99.86	99.52	99.65	99.66	99.72	100	100	99.52	99.52	99.52	100	99.52
11	ON255875.1	*Pseudomonas sp.* 20TX0180	97.16	97.42	97.23	98.40	99.86	99.86	100	100	100	100	100	99.86	99.86	99.86	100	99.79
12	ON359917.1	*Pseudomonas aeruginosa* NCTC 13628	97.41	97.60	97.39	98.56	100	99.93	100	99.86	99.59	99.52	99.86	100	100	100	100	99.93
13	ON791802.1	*Pseudomonas aeruginosa* LG-1	97.41	97.60	97.39	98.50	100	99.93	100	99.86	99.59	99.52	99.86	100	100	100	100	99.87
14	OM123592.1	*Pseudomonas aeruginosa* PJ078C9	97.41	97.60	97.39	98.50	100	99.93	100	99.86	99.59	99.52	99.86	100	100.00	100.00	100	99.87
15	NR_117678.1	*Pseudomonas aeruginosa* DSM 50071	97.34	97.60	97.39	98.62	100.	99.87	99.93	99.79	99.65	99.52	99.79	99.93	99.87	99.87	100	100
16	-	BM02	97.28	97.55	97.49	98.60	100	100	100	100	100	100	100	100.	100	100	100	100

Table S2. Analysis of variance (ANOVA) of the 23 full factorial designs for biosurfactant produced by *Pseudomonas aeruginosa* BM02.

Factor[a]	Sum-Square	Degrees of freedom	Mean squase	F	p-Value
X_1	1711.12	1	1711.12	95.0625	0.065067
X_2	7260.13	1	7260.13	403.3403	0.031673*
X_3	4371.13	1	4371.13	242.8403	0.040797*
$X_1.X_2$	6903.13	1	6903.13	383.5069	0.032480*
$X_1.X_3$	4005.12	1	4005.12	222.5069	0.042615*
$X_2.X_3$	3160.13	1	3160.13	175.5625	0.047956*
Lack of fit	4432.15	2	2216.07	123.1153	0.063599
Pure Error	18	1	18		
R^2	0.86033				
R^2_a	0.58098				

*Statistically significant results, p value <0.05; [a]X_1 = pH; X_2 = temperature (°C); X_3 = glycerol content.

Fig. S1. Phylogenetic tree of 16S rRNA sequences from BM02 and *Pseudomonas aeruginosa* and *Pseudomonas* sp. obtained from Genbank (NCBI). *P. aeruginosa* DSM 50071 was used for rooting the cladogram.

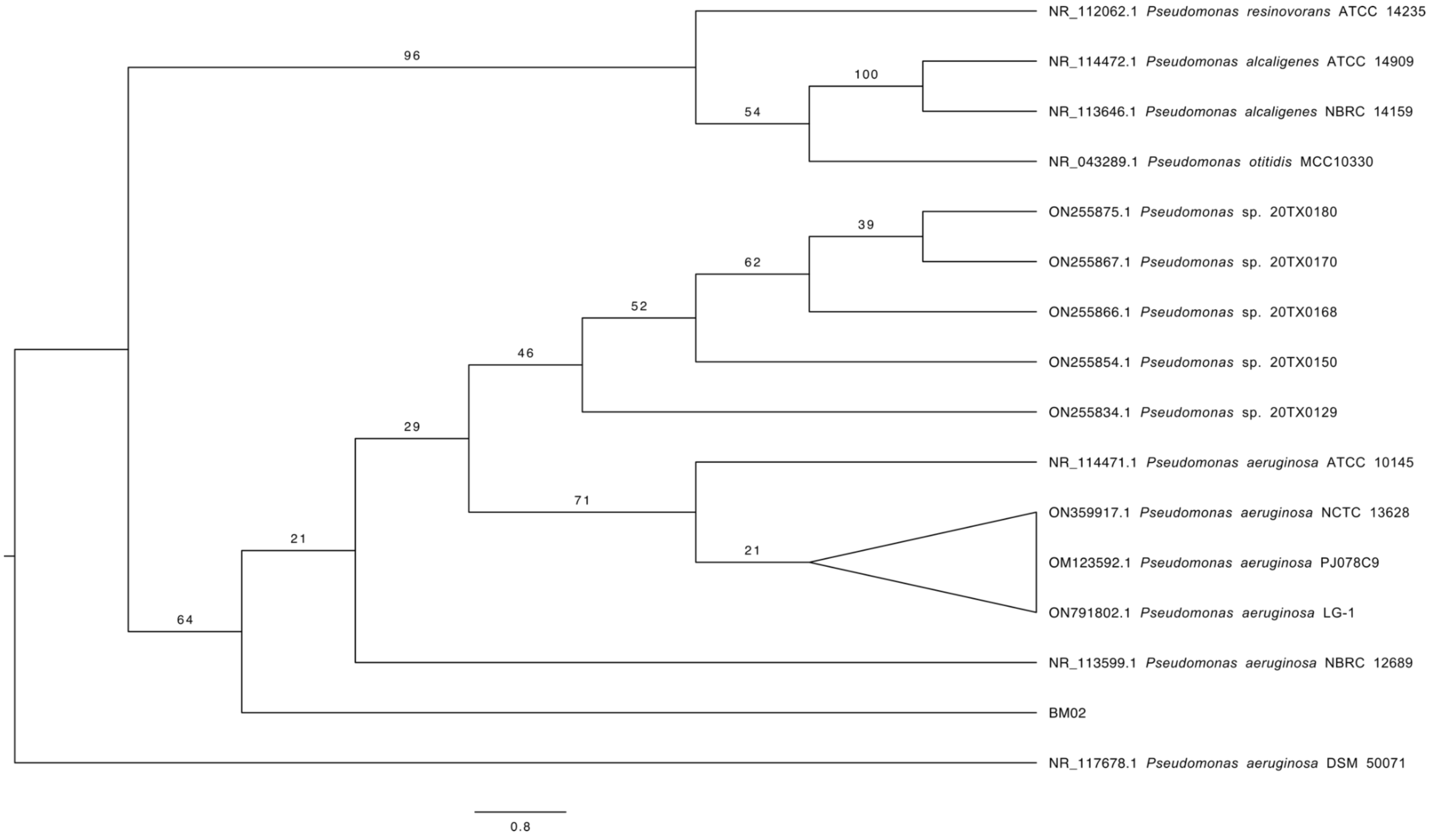

Fig. S2. Infrared absorption spectrum (FT-IR; 4000–400 cm^{-1}) of the biosurfactant produced by *Pseudomonas aeruginosa* BM02.

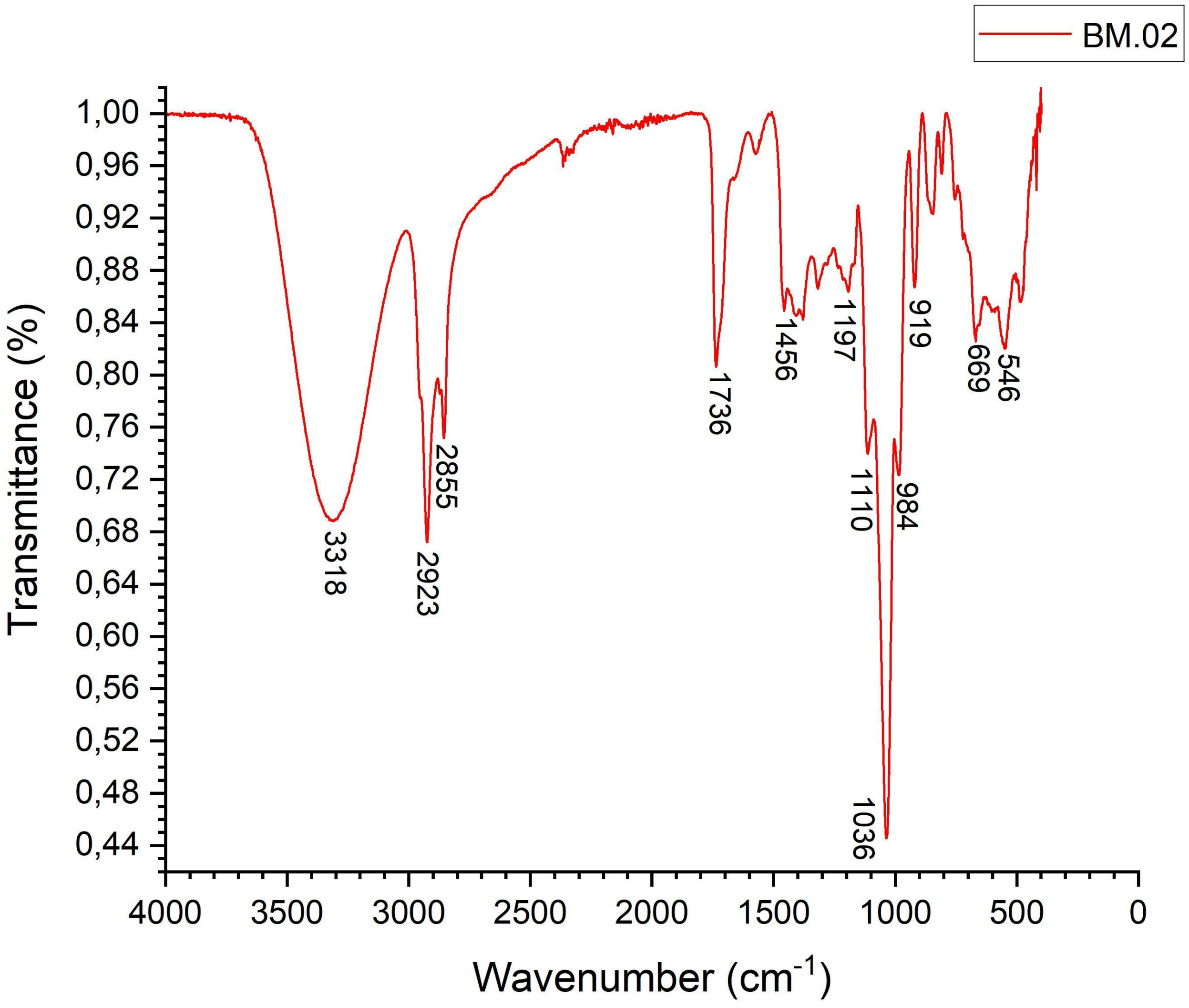

Fig. S3. ^{1}H NMR spectrum (400 MHz, 25 °C) of the biosurfactant produced by *Pseudomonas aeruginosa* BM02 (solvent: $CDCl_3$).

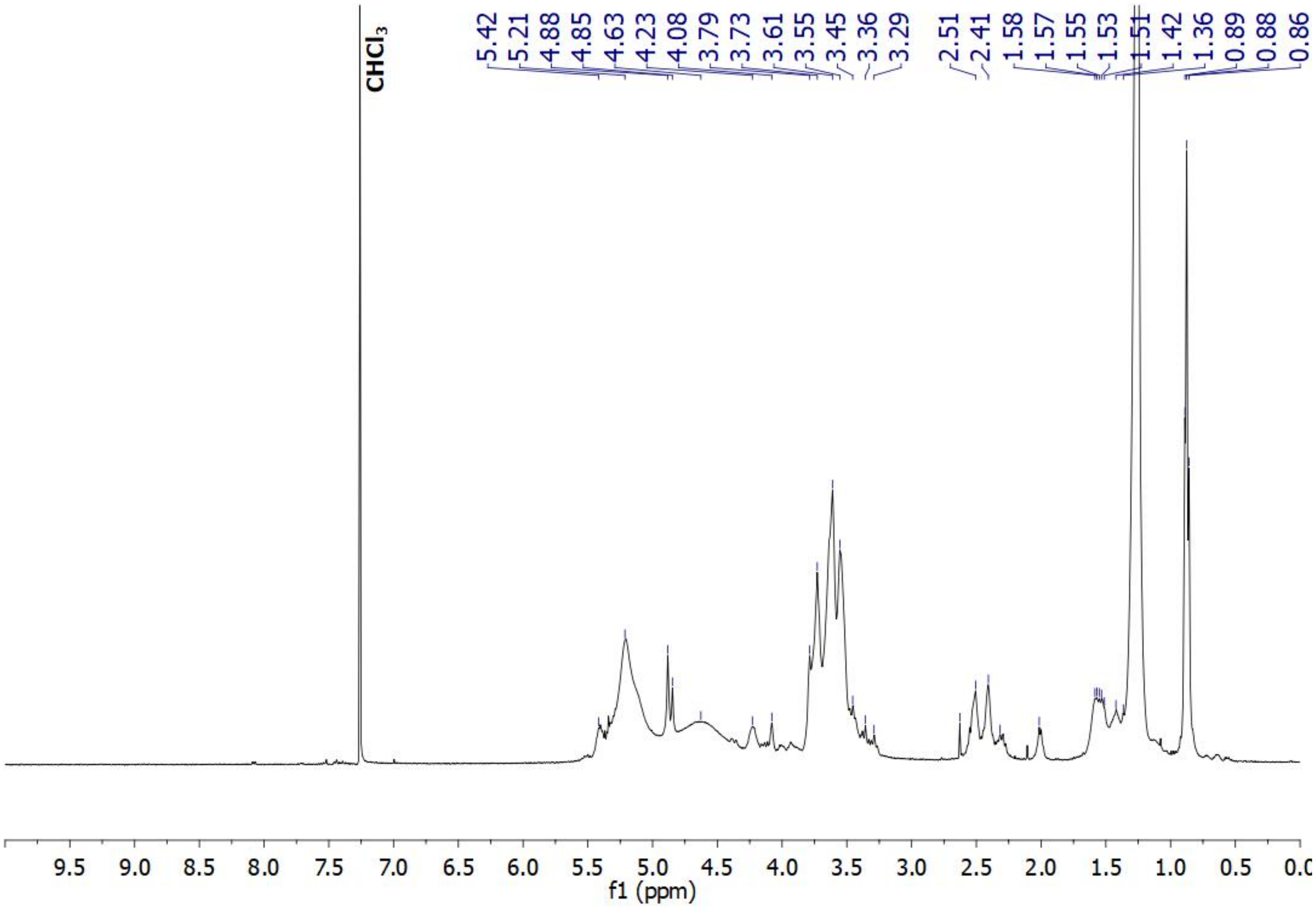

Fig. S4. ESI-MS spectrum showing the *m/z* values for the different rhamnolipid congeners present in the biosurfactant produced by *Pseudomonas aeruginosa* BM02 (A) and an expansion of the spectrum between 310 and 385 *m/z* (B).

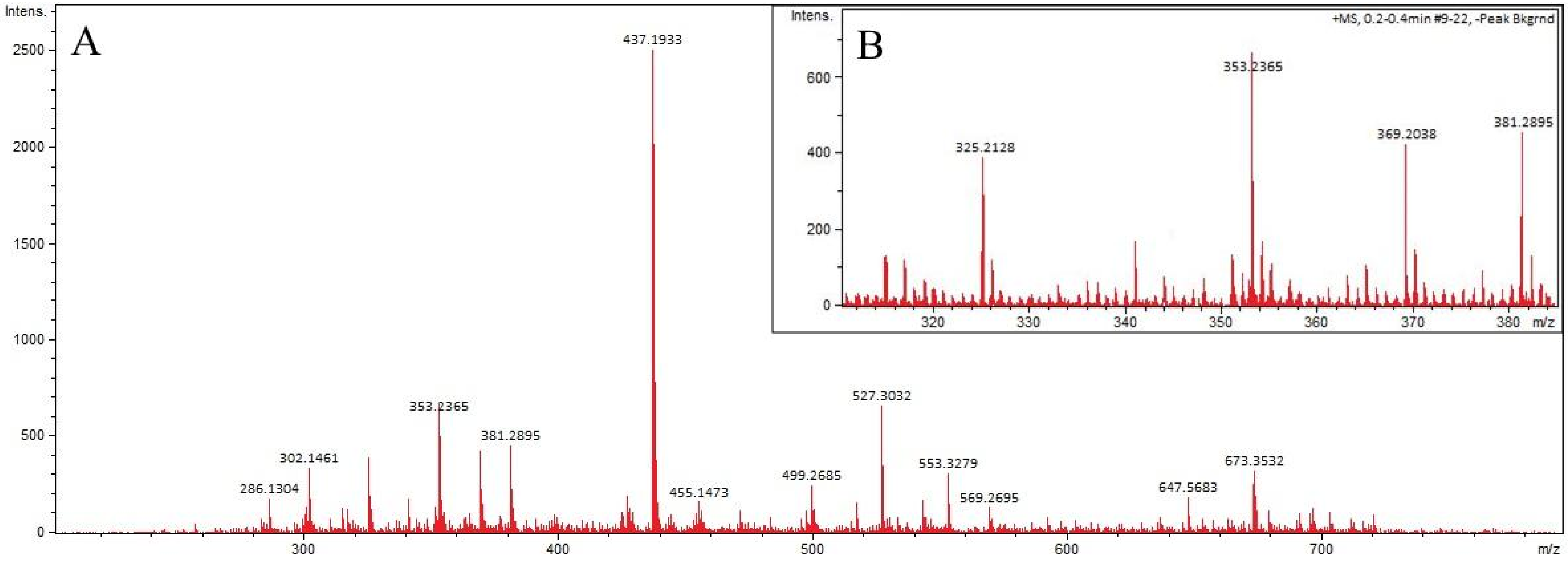

Supplement: Supplementary file 1 — Supplementary Information. [file 41598_2024_54828_MOESM1_ESM.pdf]
